# Ethno-racial categorisations for biomedical studies: the fair selection of research participants and population stratification

**DOI:** 10.1007/s11229-024-04769-8

**Published:** 2024-10-02

**Authors:** Tomasz Żuradzki, Joanna Karolina Malinowska

**Affiliations:** 1 Institute of Philosophy & Interdisciplinary Centre for Ethics, Jagiellonian University in Kraków, ul. Grodzka 52, Kraków, 31-044 Poland; 2 Faculty of Philosophy, Adam Mickiewicz University, ul. Szamarzewskiego 89C, Poznań, 60-568 Poland

**Keywords:** Clinical research, Race, Methodology, Research ethics, Reference class problem, Subgroup analysis, Population categorization

## Abstract

We argue that there are neither scientific nor social reasons to require gathering ethno-racial data, as defined in the US legal regulations if researchers have no prior hypotheses as to how to connect this type of categorisation of human participants of clinical trials with any mechanisms that could explain alleged interracial health differences and guide treatment choice. Although we agree with the normative perspective embedded in the calls for the fair selection of participants for biomedical research, we demonstrate that current attempts to provide and elucidate the criteria for the fair selection of participants, in particular, taking into account ethno-racial categories, overlook important epistemic and normative challenges to implement the results of such race-sorting requirements. We discuss existing arguments for and against gathering ethno-racial statistics for biomedical research and present a new one that refers to the assumption that prediction is epistemically superior to accommodation. We also underline the importance of closer interaction between research ethics and the methodology of biomedicine in the case of population stratifications for medical research, which requires weighing non-epistemic values with methodological constraints.

According to a systematic review and meta-analysis, cross-sectional studies show that the combined prevalence of mortality rates from COVID-19 during the pandemic in the US was highest among African American individuals (277.15 per 1000 patients), followed by Hispanic/Latino individuals (213.34 per 1000 patients), White individuals (173.38 per 1000 patients), and Asian individuals (80.4 per 1000 patients) (Magesh et al., 2021).^1^ Despite this African American and Hispanic/Latino populations have been disproportionately underrepresented in clinical trials on treatment for COVID-19 (Chastain et al., 2020). For some scholars, this is a clear mark of some kind of unfairness (Spector-Bagdady et al., 2022; Wilkins et al., 2020). Indeed, at first sight, it may seem that if, for example, individuals classified, usually based on self-declarations, as African American systematically differ in the US from, e.g., individuals classified as Asian in their health outcomes, such ethno-racial categorisation should be somehow taken into account, for both epistemic and ethical reasons, in the regulatory requirements regarding designing, conducting, and interpreting biomedical research with human participants.^2^ For many regulators and scholars, it may seem reasonable to require researchers to recruit ethno-racial subpopulations in specific proportions while designing trials with human participants (or at least not to exclude disproportionally the representatives of some populations) and to report any differences between ethno-racial subpopulations while presenting results of such trials (this is, so-called, the fair subject selection requirement). It is so because clinical research results are supposed to be used to make predictions about the effectiveness of medical interventions outside the study setting, that is, they are expected to be externally valid, and are used to create diagnostic algorithms and practice guidelines based on ethno-racial classifications (Vyas et al., 2020; cf. Fuentes et al., 2024 who shows that some of these algorithms and guidelines may rely on questionable or mistaken evidence). Unfortunately, the fair subject selection requirement, which seems to be the main justification for gathering ethno-racial statistics about human participants, is under-researched and many scholarly literature or regulatory documents do not elaborate on what makes a selection “fair” other than enumerating some examples of distributing benefits and burdens.

Therefore, referring both to discussions in research ethics and methodology of biomedicine, we claim that ethno-racial stratifications (e.g., as defined in the US legal regulations) should not be used as default as it is now. We argue there are no scientific or social reasons to routinely require gathering ethno-racial data (no matter whether self-declared or established in other ways) if the scientists or regulators themselves have no hypotheses as to how to connect this type of categorisation with any biological (inc. genetic), environmental or social mechanisms that could explain alleged inter-racial health differences and guide treatment choice. The reason for this is that diagnostic algorithms and practice guidelines in medical practice (which are based on ethno-racial categorisation) make sense only if they are useful in designing an intervention that aims to reduce the burden of disease. To design such an intervention one (usually) needs to previously hypothesise how it will act, that is, to hypothesise about its mechanism. Thus, we refer to mechanistic approaches in the philosophy of science, which claim that a successful explanation of a phenomenon – in particular in broadly understood biology and medicine, but also in the social sphere – involves describing the mechanism that produces this phenomenon (Machamer et al., 2000; for the context of ethno-racial categories, see: Kalewold, 2020). In the case of ethno-racial categorisation, as defined in the US legal regulations, we assume that there is no one universal mechanism responsible for racial differences in health, although the current US stratification may suggest that there is. Moreover, our main negative argument which assumes that prediction is (usually) epistemically superior to accommodation suggests that current institutional requirements in the US may encourage researchers to corroborate spurious hypotheses about alleged ethno-racial differences in health outputs and responses to treatment options.

However, we do not propose to exclude or ban using ethno-racial categories from designing, conducting, interpreting, and implementing research with human subjects and we do not deny that either the current US or some other ethno-racial classification be sometimes useful for research on some disorders or, for instance, on health inequalities. We propose that regulators require researchers who want to stratify the population with some ethno-racial criteria to justify in advance their stratification choices and to provide in advance hypotheses as to how to connect their choice of ethno-racial stratification with any mechanisms that could explain alleged interracial health differences and guide interventions regarding treatment choice. Since diagnostic algorithms and practice guidelines in medical practice only make sense if they successfully predict potential effective treatments to mitigate the estimated risk, the knowledge of mechanism, and therefore knowledge of potentially direct causal factors, is crucial.

Our positive proposal can be practically implemented in a similar way as the requirement of registering pre-analysis plans, which is widely used to mitigate biases present in research practice (Hitzig, & Stegenga, 2020). Thus, our proposal can be seen as, on the one hand, a criticism of the theoretical shortcomings of ‘the inclusion-and-difference paradigm,’ which requires the inclusion of members of various (including ethno-racial) groups generally considered to have been underrepresented previously as participants in clinical studies (Epstein, 2007), and, on the other, as an expression of some nuanced view in the philosophically-oriented discussion between conservationism and eliminativism about race in medical practice, and in particular about the categorisation of populations for biomedical studies (for general discussions on the problem whether race is a scientifically valuable biomedical research variable, see Andreasen, 2008; for a recent defence of eliminativism, see Perez-Rodriguez, & de la Fuente, 2017; and for a recent defence of conservationism, see Lorusso, & Bacchini, 2023).

The paper is divided into three main sections. The first reviews (in the historical context) the principles by which investigators select trial participants and design their inclusion and exclusion criteria, in particular, the principle of fairness in research with human subjects as well as analyses the most common criticism of using racial stratification in biomedical research and healthcare. The second introduces a novel argument that refers to discussions on why prediction is epistemically superior to accommodation and refers to mechanistic reasoning linking the intervention and outcome shared in the study and target populations. Finally, in the conclusions, we present a proposal on how to implement our approach in practice.

## The principle of the fair selection of research participants

### The institutional embedding

The US is the only major country that explicitly enlists legal requirements regarding gathering ethno-racial data in biomedical research.^3^ First, the US regulations enumerate specific ethno-racial categories that must be included in research; second, they describe conditions when trials must be designed specially to accommodate differences between such subpopulations; third, they present labelling guidelines for novel drugs regarding including ethno-racial data. We call it race-sorting practice, which is part of a broader phenomenon of ‘racialized medicine’ (Kahn, 2012).

The US NIH Revitalization Act, following the Belmont Report (BR) that identified ‘justice’ as one of three major ethical principles that should govern research with human subjects, mandated the inclusion of a sufficient number of women and members of “minority groups” as subjects in clinical research “such that valid analyses of differences in intervention effect can be accomplished” (National Commission, 1978; for the historical context of the BR see Nukaga, 2019). The NIH Guidelines published in 1994 additionally required that for Phase III clinical trials researchers must review the evidence to show whether clinically important gender or race differences in the intervention may be expected or not (NIH, 1994, 2001). A current version of this requirement (that applies to all NIH-conducted or supported NIH-defined Phase III clinical trials) specifies that results of valid analyses by race should be submitted to Clinicaltrials.gov “if the data from prior studies strongly support the existence of significant differences of clinical or public health importance in intervention effect based” (NIH, 2017). Similarly, the Food and Drug Administration (FDA), referring to differences in response to medical products (both intrinsic, e.g., genetics, metabolism, elimination; and extrinsic, e.g., diet, environmental exposure, sociocultural issues), also issued non-binding guidance on the collection of race and ethnicity data in clinical trials that concern both data on enrolment of racial subgroups and analyses of safety and effectiveness data by racial subgroups (FDA, 2005, 2016).

All these documents define ‘minorities’ as a subset of the US population “which is distinguished by either racial, ethnic, and/or cultural heritage,” referring to Office of Management and Budget (OMB) Directive No. 15 that distinguishes two ethnic categories (Hispanic/Latino or no Hispanic/Latino)^4^ and five racial categories (American Indian or Alaska Native; Asian; Black or African American; Native Hawaiian or Other Pacific Islander; White).^5^

In contrast to the US, International Ethical Guidelines for Health-Related Research Involving Humans recognizes the principle of fair selection of research participants and declares that equity may require special efforts to include members of underrepresented populations, but then enumerates only children, women, and pregnant women, but not ethno-racial data (CIOMS, 2016). Similarly, recent regulations on research with human subjects issued by the European Union (EU) neither directly mandate the inclusion of ethno-racial data nor give any examples referring to race, but only to gender or age (Regulation, 2014). The definition of ‘sensitive personal data’ in the EU’s General Data Protection Regulation (GDPR) includes “personal data revealing racial or ethnic origin,” and the document goes on to say that “the use of the term ‘racial origin’ does not imply acceptance by the EU of theories that attempt to determine the existence of separate human races”. The European Medicines Agency (EMA), the counterpart of the US FDA, has neither provided any formal regulations about ethno-racial data nor has it signaled any commitment to such data in some informal ways (e.g., through commentaries). Moreover, in many continental European countries, official requests about someone’s race are illegal (e.g., in France: ACT 78 − 17, 1978) and may be treated as rude or insulting (Oppenheimer, 2008). A recent review shows that 19 out of 41 European countries analysed officially do not collect data even on ethnicity (e.g., France, Germany, Italy), although in practice researchers outside the US often use ethno-racial language informally (Simon, 2012; Bartram et al., 2023; Malinowska & Żuradzki, 2023b).

The most important, however, is that although ethno-racial data are sometimes collected outside the US, EU regulators do not formally require researchers to ensure that participant enrollment is sufficiently inclusive in terms of racial or ethnic diversity, and therefore, such data basically cannot be used to evaluate population health (Simon, 2017; Villarroel et al., 2019). Although many scholars claim that in the EU ethno-racial data are ‘tabooed’ (M’charek, 2014), a recent study shows that, on the contrary, the ethno-racial concepts are being used by some EU institutions, in particular, by the EMA (Mulinari et al., 2021; Mulinari & Bredström, 2024; HLGNED, 2021).^6^ A recent study on drugs approved between 2014 and 2018 by the FDA and EMA showed that almost 90% of novel drugs approved by the FDA and 75% of novel drugs approved by EMA had at least one race/ethnicity labelling statement. In this period no drug was specifically indicated or restricted to a racial/ethnic population, as previously dinitrate/hydralazine in the US (sold under the brand Bidil, see Kahn, 2012). The authors of this study conclude that “much of the race/ethnicity labelling seems to primarily reflect the [US] policy emphasis on the inclusion of racial/ethnic groups in the trials” (Mulinari et al., 2021).

### The justifications of race-sorting practice: epistemology and ethics

What were the main reasons for introducing the legal requirement of race-sorting practice in the US? The common justifications provided in the US legal documents (FDA, 2005, 2016; OMB, 1995), and – in a more nuanced form – scholarly texts refer to ‘justice’ or ‘fairness’ in the selection of research participants. Usually, such justifications refer to the need to fill gaps in knowledge regarding representatives of minorities and to capture either biological (including genetic) differences between subpopulations and/or the effects of racism on people’s health (epistemic aim). The main rationale for filling such gaps in knowledge is to advance more precise interventions tailored to specific subpopulations and to achieve, in the long run, more equal healthcare (ethical aim). The race-sorting practice is part of ‘the inclusion and difference paradigm’ which aims to include members of groups underrepresented in previous research as participants in clinical studies to measure differences across these groups concerning “treatment effects, disease progression, or biological processes” (Epstein, 2007). A standard assumption behind such an approach is that: “self-identified races turn out to be excellent proxies for complex variation in risk-related environmental, psychological, cultural and social factors that it would be hard to fully account for in any other way” (Lorusso, & Bacchini, 2015).

#### An epistemic aim

Regarding the epistemic aim, the advocates of the race-sorting practice treat the US racial stratification as useful for refining sampling in medical research, particularly in clinical trials, where it’s important to have a proper representation of human diversity in both biological and/or social sense (Burchard et al., 2003). A standard clinical trial involves administering a potential medical intervention to an experimental group, administering a placebo or competitor intervention to a control group, measuring the parameters of the participants, comparing the values of those parameters between the groups, and “inferring a general effectiveness capacity from the difference in values of the parameters between the groups” (Stegenga 2015). However, human populations are heterogeneous in terms of variables that might be relevant, but which cannot be manipulated by the researchers (race and ethnicity, among others). When these variables are not balanced among the study groups, they may become confounding factors. An obvious way of controlling for confounders is by allocating participants to the study groups such that such confounders are balanced among the groups, and similarly represented within each. To realize this aim the representatives of relevant subpopulations must be included in a trial in the first place.

It is expected that refining sampling for trials is important for diagnoses and treatments, since the results of clinical trials, as it is commonly assumed, can be used to infer the effectiveness of the medical intervention in question.^7^ The assumption that extrapolation of trial results is more reliable if participants of trials are more similar to patients in the broader population may seem obvious at first sight and is commonly accepted in the literature (Williamson, 2019, p. 51). But which subpopulations are relevant for ‘similarity’? Let’s see this on an example of why ethno-racial categories seem relevant. Stegenga (2015) worries that in the RECORD trial, which tested the safety of an antidiabetic drug rosiglitazone, “99% of the subjects in the trial were Caucasian (despite the fact that the trial was performed in dozens of countries)”. The worry seems relevant because in common opinion belonging to some ethno-racial categories is a risk factor for type-2 diabetes (T2D), although it is not clear what exactly causes the racial/ethnic differences in incidence of T2D, in particular, “the relative contributions of genetic and environmental factors to such differences are largely unknown” (Golden et al., 2019). In such a case (as well as in our initial example about COVID-19 and many others), one might think that inclusion in trials of groups most at risk from T2D (and so those who stand to benefit most) is methodologically desired (and because of this reason may be required by the regulators) even without any prior hypotheses about the mechanism. Therefore, it may seem that the prevalence of ethno-racial health disparities which are commonly discussed in the US context, may seem to justify the race-sorting requirement. In Section 2, we will present a more detailed argument as to why this practice, while apparently justified in concrete cases, may lead to the production of spurious generalizations about ethno-racial subpopulations when used on a large scale.

#### An ethical aim

The second, ethical aim expressed in the fair subject selection requirement is, in its vague form, commonly accepted in medical journals, regulations, and guidelines. Here is a recent example concerning recruitment to clinical trials: “Ignoring the terms ‘race’ and ‘ethnicity’ risks disregarding real social stratifications and inequities that exist” (Candelario et al., 2023). However, the requirement seems under-researched (but see: MacKay, & Saylor, 2020; MacKay, 2016; London, 2022; Weijer, 1996; Kahn et al., 1998; Meltzer, & Childress, 2008; Żuradzki, 2020). The widely cited review article *What makes clinical research ethical?* published in 2000, in which authors synthesized the codes, declarations, and the relevant bioethical literature, lists “fair subject selection” among the seven most common requirements of ethical clinical research (Emanuel et al., 2000). However, many papers or documents do not elaborate on what makes a selection “fair” other than enumerating some examples of distributing benefits and burdens, do not specify the criteria for delineating groups or classes of persons, nor do they define “under-represented populations” other than based on sex, age, and as in the US, ethno-racial categories. For example, the recently published book *The Ethics of Research with Human Subjects* contains the section “Subject selection,” but the problem is discussed only on one page, which is in sharp contrast with the other principles of research discussed in this book (Resnik, 2018). An entry about fairness in *The Oxford Textbook of Clinical Research Ethics* concludes: “Determining what exactly counts as proportional or fair representation in research remained an unanswered question” (Meltzer & Childress, 2008).

One can interpret the fair subject selection requirement in a narrow way as serving to break down the barriers that still affect some populations underrepresented in clinical trials, independently of filling a knowledge gap on minorities’ health. However, it seems that fairness in the fair subject selection requirement goes beyond mere expressivist or symbolic issues, and the trials with human participants are understood as aimed at distributing some substantial good that should be fairly allocated among the members of some population: “In human research ethics, whether the selection of research participants involved in a study is fair or equitable is a distributive question” (Resnik, 2022; p. 503 cf. MacKay, 2016 & Spector-Bagdady et al., 2022). What kind of good may be distributed? The simplest answer would be that the trial participants will receive some direct benefits, e.g., a more effective treatment (Spector-Bagdady et al., 2022, p. 52).

A more nuanced answer is discussed in a recent paper, a rare attempt at a deeper analysis of the principle of fair subject selection problem. It notes that this principle should be understood as “a bundle of four distinct sub-principles” (i.e., fair inclusion, burden sharing, opportunity, distribution of third-party risks) that may conflict with each other, and each of them concern different subpopulations (MacKay & Saylor, 2020). The first (“inclusion”), which is the most important from our perspective, tracks fairness understood as a set of rules concerning the distribution among members of some population of the expected benefits that are supposed to stem from the growth of future generalizable knowledge (“The selection of research participants must be sufficiently inclusive to ensure that the research in question fairly benefits members of society.”) This growth may result from the fact that sometimes people differ “in ways that are relevant to the disease or intervention,” so the extrapolation of the research result will depend “on the nature of the participants” enrolled in the research. Therefore, as MacKay and Saylor argue, without respecting the fair inclusion principle knowledge produced in research might not be generalizable across all groups, and members of some excluded groups may not ‘benefit’ in the future to the same extent as others that have been included. The authors, like many other authors, assume that ‘racial groups’ (as understood in the US) represent an obvious way to categorise populations: “Governments have an obligation to ensure that participant enrollment is sufficiently inclusive to produce knowledge that is truly generalizable across racial groups.” However, neither they present any argument as to why racial categorisation is especially important for knowledge generalization in comparison with other possible population stratifications, nor do they describe methods to measure expected benefits from the growth of generalizable knowledge.

In Section 2, we will present a new argument that directly refers to two crucial notions characteristic of the principle of fair subject selection: “generalizability” (of knowledge) and “benefits” (from knowledge). We will demonstrate that the situation is much more complicated than the proponents of the principle of fair subject selection assume. Before that, in the next subsection, we briefly enumerate some standard arguments against the race-sorting practice to demonstrate how our proposal develops existing debates.

### The standard arguments against using race-sorting practices

First, racial categories seem not to be reliable bases for meaningful comparisons between groups, since race or ethnic classifications – like those used in the US – “are very often a mixture of folk racial categories based on phenotypic features like skin color (a distinction between ‘white’ and ‘black’), historical contingencies, including the imperial and colonial past of some Western countries (‘Hispanic/Latino’ in the US; ‘black African’ and ‘black Caribbean’ in the UK classification), and current political borders (a distinction between ‘white Irish’ and ‘white British’ in the UK classification)” (Malinowska & Żuradzki, 2023b). The same concerns medical guidelines that sometimes use ethno-racial classifications containing some arbitrary distinctions within official OMD racial/ethnic groups, e.g., Chinese Americans, South Asians, and East Asians (e.g., American College of Cardiology, 2018). Although the FDA (2016) states that the main reason for the use of the standardized OMB race and ethnicity categories is to ensure consistency in demographic subset analyses, the documents themselves use other categories (e.g. Chinese) that are assumed to roll up to the main OMB categories. There are no international or universal principles for reporting and using demo-geographic categories in research, so comparisons of different studies or data are often impossible (López et al., 2017; Huddart et al., 2019; Zhang & Finkelstein, 2019). This regulatory messiness stems from the fact that ethno-racial categories (as defined in the US regulations) are not ‘discovered’ by scientists, they are not “natural kinds” but rather are constructs built on the process of idealization of certain populations to obtain specific scientific, political, economic, or cultural goals. Therefore, it is not surprising that although researchers often incorporate these categories in their study, they are often not able to define or operationalize these concepts precisely, and some of them openly admit that they are not confident in their ability to distinguish between the terms ‘race,’ ‘ethnicity,’ and ‘ancestry’ (Popejoy et al., 2020; Malinowska, Żuradzki, 2023).

Second, as some scholars argue, even if one wanted to improve somehow current racial stratifications, there are multiple, equally ‘scientifically legitimate’ ways of carving up the population for research, especially when it comes to social characteristics (Ludwig, 2016). This is so because each individual can be assigned to an infinite number of categories depending on the characteristic that serves as its determinant. In biomedical research, these categories are often called subgroups or reference classes. This problem is particularly visible in the case of polygenic risk scores. Some sophisticated clustering algorithms can reveal elements of genetic population affinities. However, such algorithms do not produce a privileged set of clusters that are ‘right’ in all health-related cases. Instead, “there are better and worse ways of cutting the pie for particular purposes” (Kaplan, & Fullerton, 2022).

Third, the automatic use of racial classifications as reference classes may divert researchers’ attention from other possible data stratifications and selection of research subjects, as well as influence their methodological choices and analysis of results (Malinowska & Żuradzki, 2023b). For instance, some alleged differences between disease manifestations and race/ethnicity might ‘disappear’ after controlling a socioeconomic status that very often drops out of sight of researchers (Lee, 2009; Singh & Steeves, 2020; Fujimura & Rajagopalan, 2011).

Fourth, encouraging health disparities research based on ethno-racial classification without explicitly mentioning its connection with racism contributes to the strengthening of stereotypes about ethno-racial categories and racialised groups (Yearby, 2021). In particular, adopting racial stratification may in practice lead to unjustified “medicalizing” or “biologizing” races, that is, ascribing the results of racism or social inequalities to some unspecified biological (including genomic) differences as if skin color caused some unique contributions to physiologic differences (see, e.g., Fernández Pinto, 2018). Although the newest guidelines by medical institutions and journals, e.g., the newest guidance on the reporting of race and ethnicity published by JAMA (Flanagin et al., 2021), highlights that “race and ethnicity are social constructs, without scientific or biological meaning”, many scholarly papers biologize ethno-racial categories, e.g., race and ethnicity are commonly used as a proxy for genetic lines. However, there is no solid argument for this practice because the genotype-phenotype relationship is not linear (or, in other words, variability patterns captured by ethno-racial categories at the phenotypic level (if any) do not have a linear correspondence with variability patterns at the genotype level) (Malinowska, & Serpico, 2023).

For example, a review paper on racial differences in drug disposition and response published in *JAMA* defined ’race’ as referring “to a group of people who share common biological characteristics that distinguish them from other groups” (Ramamoorthy et al. 2015). Under this interpretation stating that someone belongs to a white ‘race’ picks out a natural kind (similar, for example, to ‘oxygen’), not a socially constructed kind (similar, for example, to a ‘student’). A paper discussing racial disparities in outcomes among COVID-19 patients described differences in the odds of hospitalization between races after adjustment for age, sex, comorbidities, and income. The authors speculated that: “One hypothesis is that there may be some unknown or unmeasured genetic or biological factors that increase the severity of this illness for African Americans” (Azar et al., 2020; see also Anyane-Yeboa et al., 2020, cf.Malinowska & Żuradzki, 2023b; Jabloner & Walker, 2023).

This problem is central to our argumentation: we assume that the requirement to report ethno-racial data from every clinical trial with human participants leads to the tendency to formulate a relatively (in comparison to situations where there would be no such requirement) high proportion of false hypotheses prior to testing. This is because such hypotheses do not refer to any prior reliable knowledge but mainly to certain formal/legal requirements. As noticed by Bird (2021) in the context of replication crisis: “failure to recognize this is to commit the fallacy of ignoring the base rate”. In other words, if researchers have a very high proportion of false hypotheses prior to testing, then there will be many false hypotheses that are apparently supported by the outcomes of well-conducted experiments.

Therefore, we treat statements such as those by Azar et al. (2020) as hypotheses formulated without any evidence about the mechanism between genetic or biological factors and the severity of COVID-19 illness. One can provide an infinite number of similar baseless hypotheses about the relation between the severity of C-19 illness and “some unknown or unmeasured genetic or biological factors.” Moreover, the use of the word ‘unmeasured’ in this particular example may suggest that his hypothesis is ad hoc if one understands ‘unmeasured’ as not susceptible to any empirical verification.

In response to the above arguments against using race-sorting practices, some authors argue that: “it is time to rid medical research of the highly damaging deadweight of searching for supposed racial differences in the biological manifestations of the disease, thereby conferring importance to a single or small group of phenotypic features that have no clinical significance per se” (Perez-Rodriguez, & de la Fuente, 2017). However, according to this proposal, racial categories should be still required in recruitment for biomedical research, because termination of such a criterion, in the current situation in the US when the social ascription of a racial category may be correlated with discrimination, could “lead to misrepresentation of minorities in future research,” that is, representatives of some populations would be systematically under- or overrepresented in studies with human participants (Perez-Rodriguez, & de la Fuente, 2017). According to this proposal, which could be called “the inclusion but no-analysis paradigm,” it is not clear how to reconcile the perpetuation of racial classification in recruitment for biomedical research with the total ban on analysing race as a demographic parameter with biological consequence. Thus, our paper goes in a different direction. In the next section, we present an original argument, which not only shows why the current US regulations are problematic, but also (in contrast with arguments discussed in this section) suggests a positive proposal regarding population stratification for research with human participants.

## Prediction vs. accommodation and regulations on research with human subjects

The standard model of therapeutic prediction in medicine, the risk generalization-particularization model, distinguishes two steps in the translation of study results to particular patients (Fuller, & Flores, 2015). In the first one (generalization), translation involves transferring the group-level effect size from the trial to a target population. This step concerns the ex-ante perspective of researchers who design studies with human participants to reach conclusions that are generalizable for different subpopulations. Our argument presented in this section shows that the legal requirement in the US (which informally spills over to the rest of the world) to report effectiveness and safety data for ethno-racial subgroups leads to the production of spurious generalizations about subpopulations that are delineated on the basis of ethno-racial criteria. In the second step (particularization), translation involves the transformation of the effect size into an expected change in outcome for individual patients in the target population. This step is important to evaluate alleged benefits for patients’ diagnostic algorithms and practice guidelines, resulting from race-sorting practices regarding the inclusion of research participants, that adjust their outputs based on a patient’s ethno-racial classification (Vyas et al., 2020). If step one indeed results in spurious generalizations, the applying diagnostic algorithms and practice guidelines, which are based on such results, may distort individualized risk assessment in the case of individuals classified as members of some ethno-racial subpopulations.

### Prediction vs. accommodation in biomedical research

According to the view called (weak) predictionism in the philosophy of science one should have, all other things being equal, more confidence in a hypothesis if it managed to correctly predict some result, than if it was designed to accommodate this result. According to a strong version of this view, prediction is intrinsically superior to accommodation. The very distinction between prediction and accommodation may be visualized in the following two hypothetical scenarios (adapted from Hitzig, & Stegenga, 2020). Let us assume that in both hypothetical trials, the same sample size, treatment effects, and other relevant properties are identical.

In the first case (Scenario 1), a scientist wants to test the capacity of a substance to decrease the risk of, e.g., heart attacks. After the clinical trial is over, she analyses the data and finds no difference in the frequency of heart attacks between the group that received this active substance and the group that received a placebo. She re-analyses the data by stratifying the population by sex and again finds no difference. Then, she stratifies the population by ‘race’ into two groups (white and non-white), and again there is no difference. But, when she combines the sex and ‘race’ stratifications, she observes that not-white men in the active arm of the trial had a lower frequency of heart attacks compared to the same demographic in the placebo arm. She treats these trial results as evidence (E1) and uses it to formulate a hypothesis (H1): “This substance lowers the risk of heart attacks in not-white men.”

In the second case (Scenario 2), the scientist specifies the null hypothesis (H0) as “this substance does not lower the risk of heart attacks in not-white men” and the alternative hypothesis H2, which has the same content as H1 but is formulated in advance. Then she designs the trial to include (to both arms of the trial) only participants defined as not-white males. She indeed observes (E2) that participants of the active arm of the trial had a lower frequency of heart attacks compared to the same demographic in the placebo arm.

A popular view is that evidence E1 in Scenario 1 provides much weaker confirmation of hypothesis H1. In contrast, evidence E2 may be treated as confirming H2 more strongly in Scenario 2. This is so because evidence E2 in scenario 2 has not been used to formulate hypothesis H2 and it may count as genuinely “a use-novel prediction” of hypothesis H2 (Douglas, & Magnus, 2013). It means that even if evidence E2 had been somehow expected by the scientists before the trial, it would have not been used by them in constructing hypothesis H2. Therefore, what is important is not the order in time in which hypothesis H2 is constructed and the evidence E2 discovered, but the motivations of the scientists (or more general: the state of their mind) while constructing H2. In contrast, E1 has not been predicted by H1 (as E2 by H2), but the other way round: the scientists used E1 to design H1 (for example, it could be the case that they modified some previous hypothesis HX to H1 in such a way to accommodate E1). Thus, although in some contexts accommodated evidence can have some confirmational value, scenario 1 involves a practice that may be treated as a form of P-hacking: grouping the same data in many different ways to perform multiple statistical analyses on them (of course, the P-hacking practice may also have many different forms). Although Scenario 2 seems methodologically favorable, it may be favorable because of a variety of reasons.

First, one can expect that the reason why the scientists specified the hypothesis H2 in advance is that she had additional prior evidence, let us call it EX, in favor of H2. For example, this may suggest that the scientists formulated hypotheses (that could be at different levels of confirmation) about causal mechanisms (e.g., biological, social, environmental) by which some substance lowers the risk of a heart attack only among young not-white men (but not among others). Of course, hypotheses about a causal mechanism are not the only possible evidence in question. In particular contexts observing correlations in previous studies may also be a legitimate motive for further explanatory studies or even the reason why the scientists specified hypothesis H2 in advance without hypotheses about mechanisms. However, if the scientists have some hypotheses about mechanisms in scenario 2, they may be able to explain the phenomena in question, i.e., explain why the new drug lowers the chance of heart attacks in not-white men and may be able to manage to use this explanation to design some medical interventions. To do so one needs to know *how* the substance reduces the risk of a heart attack only among young not-white men and *why* only in this subpopulation. In contrast, in Scenario 1, the scientist does not seem to have such a hypothesis ex ante and has not explained anything: she seems to conduct some exploratory expedition to find any correlation between the new substance and some demographic parameters (no matter which ones).

The second reason refers to the probability that one would observe results like that. The subgroup analyses can be treated as another sampling of the data, and the more such sampling is performed, the larger the sample. And in the larger samples, it is more likely to observe some unusual patterns. This could be understood in an analogy to some very rare events: the probability that you, our reader, will die in a flight accident in 2024 is extremally low (even if you are a frequent participant in scientific conferences overseas), but the probability that someone will die in a flight accident in 2024 is extremally high. The same concerns subgroup analyses: the more subgroup analyses are performed (where subgroup membership may be defined either by race or by other stratifications) after some trial on a new active substance, the more probable that some of the analyses will find a positive effect. Moreover, partitioning a population into subgroups in completely arbitrary ways (e.g., based on ethno-racial criteria by default) may decrease sample sizes within groups and may decrease the statistical power of such a trial (i.e., pre-test probability that the statistical test will reject the null hypothesis, on the assumption that the null hypothesis is false). Smaller samples make it more likely that a scientist detects significant results simply due to chance, and that truly significant results remain masked by the coarse-grained noise encountered.

The third reason in favor of Scenario 2 stems from the probability of false positive results. In general, one may expect that the results of studies that follow scenario 1 are more commonly false than the results of studies that follow scenario 2. The reason is the base rate fallacy which arises when researchers focus solely on some salient piece of evidence regarding that occurrence while neglecting the rate at which occurrences of that phenomenon would occur independently of that evidence (the base rate) (Bird, 2021). In randomized controlled trials (RCT) it is commonly accepted that results are analysed by null hypothesis significance testing (NHST), with the significance level set at 5% (*p* < 0.05). It means that using NHST researchers calculate the probability that they would see the observed difference in outcome if the null hypothesis were true. If *p* < 0.05 the null hypothesis is rejected and the research hypothesis is accepted - researchers may publish the result of the study as a statistically significant outcome suggesting, for example, that some drugs lower the risk of heart attacks in not-white men.

However, this method is not perfect, and it accepts a false positive rate of 5% (one may expect that only 95 out of 100 of our studies are accurate in this respect). If the regulators, like the FDA, require “to include summaries of effectiveness and safety data for important demographic subgroups, including racial subgroups” (FDA, 2016) (even without any evidence from prior studies that there are any significant differences in intervention effect), it is analogical to the requirement of testing random hypotheses which in the overwhelming majority of cases are false. Let us assume, as a hypothetical example, that only 1 in 1000 such ‘hypotheses’ about the effectiveness and safety of new drugs are in fact true (that there are statistically significant outcomes concerning the relation between some ‘race’ understood as in the US legal documents and effectiveness or safety of some new drug). This proportion was suggested by Ioannidis (2005) for “discovery-oriented exploratory research with massive testing.” If we accept the significance level at the standard level of 0.05, we can ask if a report states that a drug is effective for some racial subgroups. In other words, what *in fact* are the chances of its being true? Certainly not 95%, because the number of true hypotheses is minuscule in the group of all hypotheses tested. The right answer is less than 2%.^8^ Even if we increase the rate of true ‘hypothesis’ about the effectiveness and safety of new drugs, the problem remains.^9^ Thus, this argument suggests that current institutional requirements in the US may encourage researchers to corroborate spurious hypotheses about races and disease or treatment options, which is relevant, for instance, in the context of diagnostic algorithms and practice guidelines that adjust their outputs based on a patient’s ‘race’ (Vyas et al., 2020).

### Population stratifications and mechanisms

One can notice some problems with the above argument. Our thesis may seem to undermine the requirement to report (without any prior hypotheses) other demographic statistics (i.e., in practice, mostly age and sex). However, there is a significant difference between racial and other demographic stratifications: while sex is usually determined by the sex chromosomes and age by the birthdate, even many of those who defend the indispensability of racial categorisations in medical research maintain that human races do not exist in any meaningful biological sense. This issue refers to the well-known problem of the reality of ‘races’ (Glasgow et al., 2019; Spencer, 2018; Winsberg, 2022). Supporters of race-sorting practice in medical research may acknowledge that races are not real in the biological sense, that is, they are not distinct biological kinds, but they still may claim that ethno-racial categories are ineliminable from the explanation, in particular, to track the health effects of racism, and should be used as a proxy for some real variables. For example, the authors of a recent defense of the thesis of “the indispensability of race in medicine” claim that race is ‘a non-referring concept’ that can “nonetheless turn out indispensable for explaining real phenomena,” in particular health-related results of racism (Lorusso, & Bacchini, 2023). They used in this context a metaphor of witchcraft, which is obviously not real (i.e., witches do not exist), but witchcraft-related practices, such as witch hunts, were quite common in some periods and influenced the well-being of those categorised as ‘witches.’ The analogical reasoning may be applied to ethno-racial categories, because even if these categories are merely social and/or legal constructs, there still may be non-accidental differences in the distribution of biologically significant properties between different such populations resulting from social interactions. The ethno-racial categories may be causally relevant not because they refer to something ex-ante biologically relevant, but because they were created as a result of social interactions and “occurred in propositional attitudes in many people’s minds that translated into physical actions on their part” (Lorusso, & Bacchini, 2023). Thus, social practices related to both witchcraft and ethno-racial categories may be scientifically studied, although the place of such categories in causal explanations requires a short comment.

In the Potential Outcomes Approach (POA) commonly used in recent epidemiology the possibility of prediction stems from specifically defined causal knowledge about the phenomena. A cause is defined in this approach as something that makes a difference and, in particular, something that humans could, in fact (Holland, 2003) or in a feasible way (VanderWeele, & Robinson, 2014) intervene on (interventionist approach to causation). Under this assumption, there is no well-specified intervention that one can perform on ethno-racial categories since one cannot change an individual’s ‘race.’ Therefore, in the strong version of this view, being classified as a representative of a particular ethno-racial category itself cannot be a cause: “Because I am a White person, it would be close to ridiculous to ask what would have happened to me had I been Black” (Holland, 2003, p. 9). However, in less restrictive versions of this view, the concept of an intervention applies even in cases where agents cannot intervene for technical, practical, or ethical reasons. In such cases what matters is how variables would respond to an intervention, no matter if such intervention is even practically possible (see: ‘in principle’ approach by Woodward, 2003). Therefore, for example, Marcellesi (2013) assumes that it is logically, nomologically, and conceptually possible to intervene hypothetically on ‘race,’ which he understands as a mixture of biological and environmental factors. To exemplify this view, he hypothesises that ‘races’ could be (i.e., it is conceptually possible) randomly assigned to embryos 30 days after conception, while the biological factors are assigned via genetic engineering and the environmental factors – by swapping embryos between mothers (Marcellesi, 2013; 655). However, whether it would make sense to treat being classified as a representative of a particular ethno-racial category in causal terms boils down to whether it would be possible to reason counterfactually about variation in race independently of variation in an individual’s other properties since in an ideal intervention variable’s value should depend only on the intervention, but not on other causes. Such a concept of ‘race’ is not realistic since different racialised groups are heterogeneous in terms of various causally relevant properties (Dong 2023; Tolbert, 2024).

The limitations of the interventionist approach to causation in the case of ethno-racial categories are also visible in the case of explaining why particular population stratification used in a study is optimal, e.g., why one uses the US ethno-racial classification or distinguishes only white and non-white subpopulations as in our hypothetical example in Sect. 2.1. What is important in clinical trials is not any type of similarity between the populations tested and the population to which the trial aims to apply, but the relevant type in the context of the studied disease (e.g. the proportion of bald participants in the trial testing the safety of rosiglitazone discussed in Section 1.2.1 seems completely irrelevant). Although it is well known that participants in a clinical trial are virtually never drawn from a random sample of the broader population (which in general consists of more people who are sicker, older, on more medications, etc.) it is not easy to provide evidence, aligned with the current standards of evidence-based medicine (EBM), that improving sampling by taking into account ethno-racial stratification by default as proposed by the US official documents indeed improves diagnosing and treatment in general or even locally in the US context. Of course, conducting an RCT only to prove that one ethno-racial stratification is more relevant than some other or no stratification at all would be highly problematic from an ethical and regulatory perspective. Cohort studies or observational studies may provide some evidence, although much weaker according to the current EMB standards. The critics of race-sorting noticed that “Whether self-reported racial identity is an adequate proxy for salient social or genetic factors must be judged against some reference standard, but for race and ethnicity it is clear that no adequate reference standard can exist” (Kaufman et al., 2021).

These problems show important limitations of the interventionist approach to causation in this case and suggest that some other approaches, in particular, mechanistic approaches, may be more relevant in this context. If one properly designs a study to test hypothesis H2 as in Scenario 2 described in Section 2.1., one may try to explain a phenomenon by manipulating not the ‘race’ itself but some other variables based on their mechanistic knowledge of the phenomenon, e.g., racism that influences the health of some populations (which may be operationalized and measured on many different ways, see Wien et al., 2023). In such a case, the explanation ultimately amounts to the elucidation of how those causal factors interact in the (social) mechanisms to produce the phenomenon under investigation shared in the study and target populations (cf. Kalewold, 2023). This may be exemplified by a well-known example of the increased mortality and morbidity of African Americans in comparison with white Americans which does not occur in the case of recent black immigrants from West Africa to the US, who have lower disease risk for some diseases (e.g., see cases of hypertension, birth weight, and premature deliveries, as well as Alzheimer’s disease analysed by Kalewold 2023). Without knowledge about the mechanism, if one would want to refer to mere correlations between different traits of self-identified racial group membership and treatment results (as in the case of BiDil, see: Kahn, 2012), one would not explain differences in disease risks and one would not explain why some interventions work specifically for some representatives of same ‘races’ (defined in one way or another) but not for others. Thus, in the case of ethno-racial categories we have a particularly compelling reason for demanding a detailed explanation of hypotheses about the mechanisms involved, given that ‘race’ is “a non-referring concept” and thus that can be reduced to some other variables, in particular, racism that influences the health of some populations, economic and health disparities, or unequal education and job opportunities, etc.

## Conclusions: population categorisation for medical research and treatment

The interpretation presented in this paper opens the possibility to more nuanced approaches to population categorisation for biomedical research and clinical practice as suggested recently by some authors who argued for the multileveled and processual conceptualisation of racialised individuals in biomedical research (Malinowska & Żuradzki, 2023a). In particular, this approach does not delineate strict racial categories but distinguishes different factors affecting and co-shaping racialisation and different pathways through which the processes of racialisation take place (phylogenetic, epigenetic, phenotypic, neuronal, environmental, socio-cultural). In the cases discussed in this paper, it would mean that a participant or a patient may be assigned by a researcher to a few different categories (also racial) depending both on the evidence available for her as well as on the researchers’ particular aims (that include both epistemic and fairness-related). More metaphorically, it would mean that an individual in a population is not represented by one particularly coloured ball from one particular urn (population) as suggested by some current approaches (Fuller, 2020), but rather the same individual may be conceptualised as a differently coloured ball in different urns which are merely heuristic tools for researchers and physicians to deal with different levels of uncertainty (evidential, classificatory, etc.). In this approach, the racial categories as proposed by the US regulation do not present a privileged set of subpopulations that are ‘right’ for all research and health-related cases. Instead, researchers should be able to cut the population differently for different research and therapeutic purposes, and establishing which process of the racialisation of individuals is relevant is part of the research questions (Ludwig, 2016).

In the US it is common to believe that self-declared racial or ethnic status is relevant for evaluating the health of subpopulations and may be treated as a proxy for biological (including genetic), environmental, or social differences. This is the position of not only governmental institutions that try to maintain race-sorting in the infrastructure of the research process and clinical practice on the level of recruitment, analysing, and reporting research as well as clinical guidelines, but also many scholars. Our proposal is not to get rid of all uses of ethno-racial classifications in research but to permit different stratification of population and to require both regulators and researchers to justify why they require (regulators) in some contexts or plan to use in some studies (researchers) one or another ‘racial’ identifications (not necessarily as understood by the current US regulations) as a variable and a proxy for anything that is biologically, environmentally, or socially relevant. Our proposal presents a more transparent way of reporting intentions to analyse race and ethnicity subgroups. When research with human participants plans to include some racial categories, authors should clearly outline in advance (i.e., in their pre-analysis plans) the biological or clinical rationale for performing such analyses (or indicate their hypothesis-generating nature) in protocols. They should also justify the subgroup choices, recognizing that the appropriate subgroup choices will depend on the particular disease studied (Liu et al., 2020). In other words, “determine your reference classes by looking to evidence of mechanisms” (Clarke et al., 2014).

One could ask who is evaluating these justifications. Our answer is simple: Nobody. This would be similar to other pre-analysis plans which are not evaluated ex-ante but just reported and may be analysed ex-post, e.g., when researchers want to register a new drug. Therefore, our proposal requires greater transparency: researchers should be more explicit about who are ‘minorities’ that are delineated from the larger population and, as the dominant view assumes, proactively included in the research, as well as, more generally, which criteria (both methodological and fairness-related) are important to stratify populations concerning recruitment for biomedical research. From a broader perspective, our proposal demonstrates the importance of the philosophy of science for bioethical and regulatory debates (Tuana, 2010).

## Footnotes

1 See also data for English hospitals which shows that among British black Africans is more than 3.7 times that of the white British population, among British Pakistanis more than 2.9 times higher, and among British black Caribbeans more than 1.8. Yet, for the white Irish, the ratio is 0.5, see: Platt and Warwick (2020).

2 The official US documents enumerate both “race and ethnicity” (with only two ‘ethnic’ categories: Hispanic/Latino or no Hispanic/Latino) and this is why we usually use the term “ethno-racial”, but for simplicity, we sometimes abbreviate this term to ‘racial’ stratification.

3 UK also collects racial data for healthcare (mixing them with some ethnic or political categorisations like the distinction between: ‘white Irish’ and ‘white British’), but there are no explicit requirements regarding these categories in biomedical research and “there is no guidance on which ethnicity categories are appropriate for use in trials”, see: Wallace (2023).

4 Defined as “Spanish culture or origin, regardless of race.”

5 It is a different question to what extend these regulations are followed in practice, see Moloney, & Shiely, 2022.

6 For a similar study that surveyed the drug labelling of new molecular entities approved by the FDA between 2008 and 2013 see: Ramamoorthy, et al. 2015.

7 For criticism of this assumption, often called simple extrapolation, that is, extrapolation from the quantitative results of clinical trials to a target population, see Stegenga, 2015; Fuller, 2021; Tresker, 2022.

8 0.001 × 0.95 / (0.001 × 0.95) + ((1–0.001) x (1–0.95) = 0.018664047151277 (i.e., about 1. %).

9 If we assume that 1 in 10 such ‘hypotheses’ about the effectiveness and safety of new drugs are in fact true, still only two-thirds of S’s hypotheses that are successful in passing the test for truth, are in fact true.
